# (6-Meth­oxy-2-oxo-2*H*-chromen-4-yl)methyl piperidine-1-carbodi­thio­ate

**DOI:** 10.1107/S1600536813028432

**Published:** 2013-10-23

**Authors:** K. Mahesh Kumar, M. Vinduvahini, N. M. Mahabhaleshwaraiah, O. Kotresh, H. C. Devarajegowda

**Affiliations:** a Department of Chemistry, Karnatak University’s Karnatak Science College, Dharwad, Karnataka 580 001, India; b Department of Physics, Sri D Devaraja Urs Govt. First Grade College, Hunsur 571 105, Mysore District, Karnataka, India; c Department of Physics, Yuvaraja’s College (Constituent College), University of Mysore, Mysore 570 005, Karnataka, India

## Abstract

In the title compound, C_17_H_19_NO_3_S_2_, the maximum deviation of atoms in the 2*H*-chromene ring system is 0.0097 (14) Å and the piperidine ring adopts a chair conformation. The dihedral angle between the 2*H*-chromene ring and the piperidine ring (all atoms) is 87.59 (8)°. In the crystal, inversion dimers linked by pairs of C—H⋯O inter­actions generate *R*_2_^2^(22) loops. Further C—H⋯O hydrogen bonds link the dimers into [110] chains and weak aromatic π–π stacking [shortest centroid–centroid distance = 3.824 (8) Å] is also observed.

## Related literature

For a related structure and the synthesis, see: Kumar *et al.* (2012 ▶).
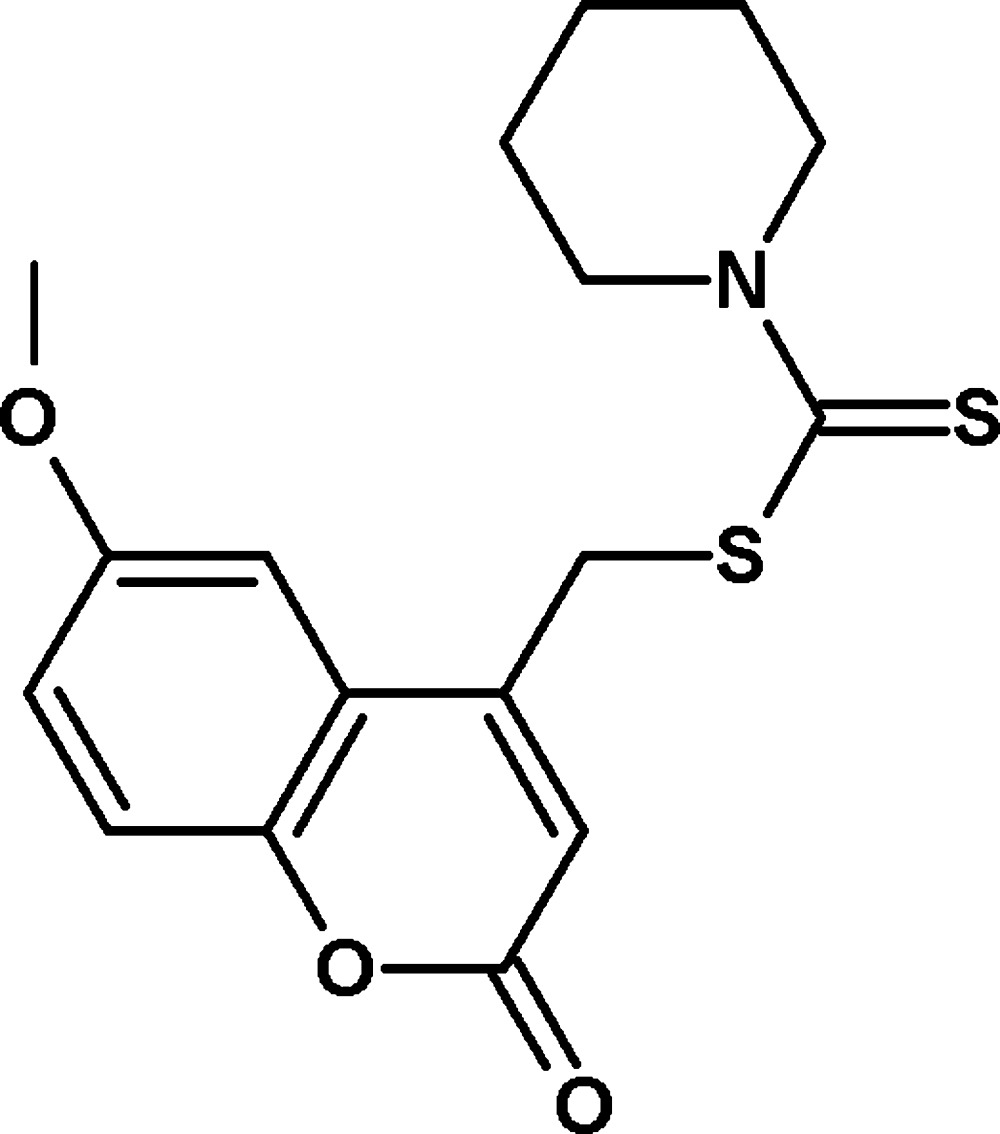


## Experimental

### 

#### Crystal data

C_17_H_19_NO_3_S_2_
*M* *_r_* = 349.45
Triclinic, 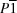

*a* = 6.9731 (2) Å
*b* = 10.2310 (3) Å
*c* = 11.9955 (3) Å
α = 92.024 (1)°
β = 90.176 (1)°
γ = 106.497 (1)°
*V* = 819.96 (4) Å^3^
*Z* = 2
Mo *K*α radiation
μ = 0.34 mm^−1^
*T* = 296 K
0.24 × 0.20 × 0.12 mm

#### Data collection

Bruker SMART CCD diffractometer
Absorption correction: multi-scan (*SADABS*; Bruker, 2007 ▶) *T*_min_ = 0.770, *T*_max_ = 1.000
17441 measured reflections
4261 independent reflections
3513 reflections with *I* > 2σ(*I*)
*R*_int_ = 0.023

#### Refinement

*R*[*F* ^2^ > 2σ(*F* ^2^)] = 0.037
*wR*(*F* ^2^) = 0.109
*S* = 1.04
4261 reflections
208 parameters
H-atom parameters constrained
Δρ_max_ = 0.28 e Å^−3^
Δρ_min_ = −0.18 e Å^−3^

### 

Data collection: *SMART* (Bruker, 2007 ▶); cell refinement: *SAINT* (Bruker, 2007 ▶); data reduction: *SAINT*; program(s) used to solve structure: *SHELXS97* (Sheldrick, 2008 ▶); program(s) used to refine structure: *SHELXL97* (Sheldrick, 2008 ▶); molecular graphics: *ORTEP-3 for Windows* (Farrugia, 2012 ▶); software used to prepare material for publication: *SHELXL97*.

## Supplementary Material

Crystal structure: contains datablock(s) I, global. DOI: 10.1107/S1600536813028432/hb7152sup1.cif

Structure factors: contains datablock(s) I. DOI: 10.1107/S1600536813028432/hb7152Isup2.hkl

Supplementary material file. DOI: 10.1107/S1600536813028432/hb7152Isup3.cml

Additional supplementary materials:  crystallographic information; 3D view; checkCIF report

## Figures and Tables

**Table 1:** Hydrogen-bond geometry (Å, °)

*D*—H⋯*A*	*D*—H	H⋯*A*	*D*⋯*A*	*D*—H⋯*A*
C19—H19*B*⋯O5^i^	0.97	2.50	3.410 (2)	157
C23—H23*A*⋯O3^ii^	0.97	2.60	3.365 (2)	136

Symmetry codes: (i) 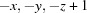; (ii) 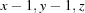.

**Fig. 1.**
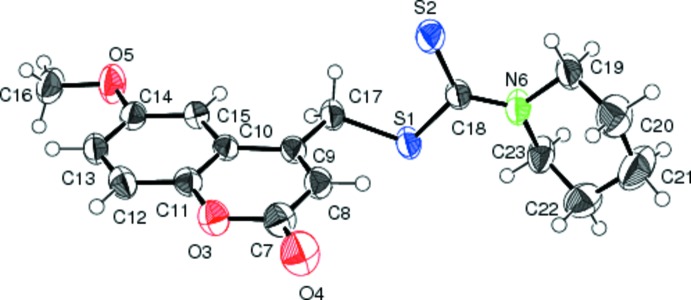
The molecular structure of the title compound. Displacement ellipsoids are drawn at the 50% probability level. Hydrogen atoms are shown as spheres of arbitrary radius.

**Fig. 2.**
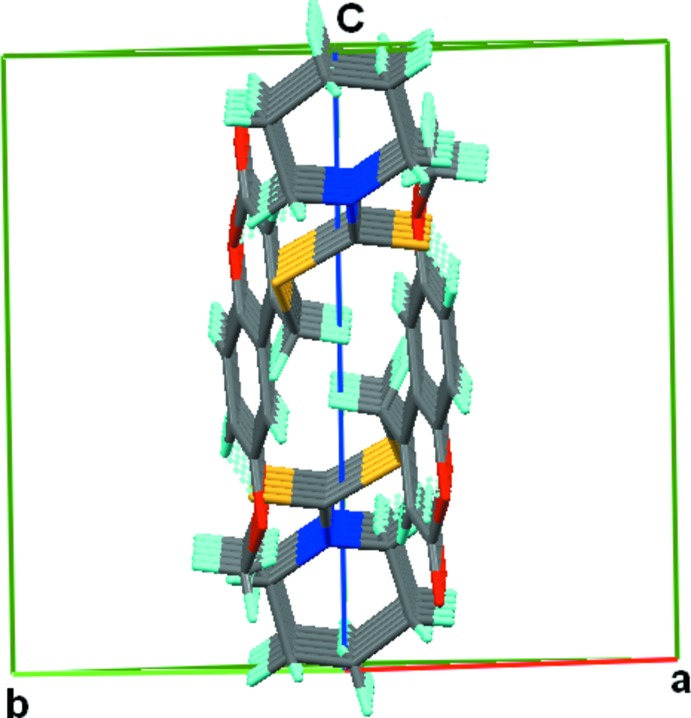
The packing of molecules.

## supplementary crystallographic information

### 1. Comment

As part of our ongoing structural studies of coumarin derivatives (Kumar *et al.*, 2012), we now describe the title compound, (I) (Fig. 1).

The 2*H*- chromene ring system is almost planar, with a maximum deviation of 0.0097 (14) Å for atom C9 and the piperidine ring adopts a chair conformation. The dihedral angle between the 2*H*-chromene (O3/C7–C15) ring and the piperidine ring (N6/C19–C23) is 87.59 (8)°. In the crystal structure, C19—H19B···O5 and C23—H23A···O3 hydrogen bonding (Table 1) and π–π interactions between the fused benzene ring (C10–C15) of 2*H*-chromene and fused pyran ring (O3/C7–C11) [shortest centroid–centroid distance = 3.824 (8) Å] occur.

### 2. Experimental

This compound was prepared according to the reported method (Kumar *et al.*, 2012). Colourless needles of the title compound were grown from a mixed solution of EtOH / CHCl_3_(V/V = 1/1) by slow evaporation at room temperature. Colour: yellowish. Yield= 79%, m.p.395 K. IR (KBr) 655 cm^-1^(C—S), 1243 cm^-1^(C=S), 1006 cm^-1^(C—O), 887 cm^-1^ (C—N), 1161 cm^-1^(C—O—C), 1719 cm^-1^(C=O). GCMS: m/e: 349. 1H NMR (400 MHz, CDCl_3_, \?, p.p.m.): 1.65(s, 6H, Piperidine-CH_2_), 3.15(s, 3H, –OCH_3_), 3.89(s, 2H, Piperidine-CH_2_), 4.20(s, 2H Piperidine-CH_2_), 4.70(d, 2H, Methylene-CH_2_), 6.45(s, 1H, Ar—H), 7.14(d, 1H, Ar—H), 7.30(s, 1H, Ar—H), 7.50(s,1*H*, Ar—H). Elemental analysis for C_17_H_19_NO_3_S_2_: C, 58.36; H, 5.42; N, 3.93.

### 3. Refinement

All H atoms were positioned geometrically, with C—H = 0.93 Å for aromatic H, C—H = 0.97 Å for methylene H and C—H = 0.96 Å for methyl H,and refined using a riding model with *U*_iso_(H) = 1.5*U*_eq_(C) for methyl H and *U*_iso_(H) = 1.2*U*_eq_(C) for all other H

### Crystal data

**Table:**

C_17_H_19_NO_3_S_2_	*F*(000) = 368
*M**_r_* = 349.45	*D*_x_ = 1.415 Mg m^−^^3^
Triclinic, *P*1	Melting point: 395 K
*a* = 6.9731 (2) Å	Mo *K*α radiation, λ = 0.71073 Å
*b* = 10.2310 (3) Å	Cell parameters from 4261 reflections
*c* = 11.9955 (3) Å	θ = 1.7–28.8°
α = 92.024 (1)°	µ = 0.34 mm^−^^1^
β = 90.176 (1)°	*T* = 296 K
γ = 106.497 (1)°	Plate, colourless
*V* = 819.96 (4) Å^3^	0.24 × 0.20 × 0.12 mm
*Z* = 2	

### Data collection

**Table:**

Bruker SMART CCD diffractometer	4261 independent reflections
Radiation source: fine-focus sealed tube	3513 reflections with *I* > 2σ(*I*)
Graphite monochromator	*R*_int_ = 0.023
ω and φ scans	θ_max_ = 28.8°, θ_min_ = 1.7°
Absorption correction: multi-scan (*SADABS*; Bruker, 2007)	*h* = −9→9
*T*_min_ = 0.770, *T*_max_ = 1.000	*k* = −11→13
17441 measured reflections	*l* = −16→16

### Refinement

**Table:**

Refinement on *F*^2^	0 restraints
Least-squares matrix: full	Hydrogen site location: inferred from neighbouring sites
*R*[*F*^2^ > 2σ(*F*^2^)] = 0.037	H-atom parameters constrained
*wR*(*F*^2^) = 0.109	*w* = 1/[σ^2^(*F*_o_^2^) + (0.0576*P*)^2^ + 0.1837*P*] where *P* = (*F*_o_^2^ + 2*F*_c_^2^)/3
*S* = 1.04	(Δ/σ)_max_ = 0.001
4261 reflections	Δρ_max_ = 0.28 e Å^−^^3^
208 parameters	Δρ_min_ = −0.18 e Å^−^^3^

### Special details

**Table:**

Experimental. IR (KBr) 655 cm^-1^(C—S), 1243 cm^-1^(C=S), 1006 cm^-1^(C—O), 887 cm^-1^ (C—N), 1161 cm^-1^(C—O—C), 1719 cm^-1^(C=O). GCMS: m/e: 349. 1H NMR (400 MHz, CDCl_3_, \?, p.p.m.): 1.65(s, 6H, Piperidine-CH_2_), 3.15(s, 3H, –OCH_3_), 3.89(s, 2H, Piperidine-CH_2_), 4.20(s, 2H Piperidine-CH_2_), 4.70(d, 2H, Methylene-CH_2_), 6.45(s, 1H, Ar—H), 7.14(d, 1H, Ar—H), 7.30(s, 1H, Ar—H), 7.50(s,1*H*, Ar—H). Elemental analysis for C_17_H_19_NO_3_S_2_: C, 58.36; H, 5.42; N, 3.93.
Geometry. All e.s.d.'s (except the e.s.d. in the dihedral angle between two l.s. planes) are estimated using the full covariance matrix. The cell e.s.d.'s are taken into account individually in the estimation of e.s.d.'s in distances, angles and torsion angles; correlations between e.s.d.'s in cell parameters are only used when they are defined by crystal symmetry. An approximate (isotropic) treatment of cell e.s.d.'s is used for estimating e.s.d.'s involving l.s. planes.
Refinement. Refinement of *F*^2^ against ALL reflections. The weighted *R*-factor *wR* and goodness of fit *S* are based on *F*^2^, conventional *R*-factors *R* are based on *F*, with *F* set to zero for negative *F*^2^. The threshold expression of *F*^2^ > 2σ(*F*^2^) is used only for calculating *R*-factors(gt) *etc*. and is not relevant to the choice of reflections for refinement. *R*-factors based on *F*^2^ are statistically about twice as large as those based on *F*, and *R*- factors based on ALL data will be even larger.

### Fractional atomic coordinates and isotropic or equivalent isotropic displacement parameters (Å^2^)

**Table:**

	*x*	*y*	*z*	*U*_iso_*/*U*_eq_	
S1	−0.11287 (5)	0.07096 (4)	0.65164 (3)	0.04415 (12)	
S2	0.27902 (6)	0.01452 (4)	0.70354 (4)	0.05410 (14)	
O3	0.28766 (17)	0.60135 (11)	0.70830 (9)	0.0468 (3)	
O4	0.2364 (2)	0.52695 (14)	0.87805 (10)	0.0691 (4)	
O5	0.2788 (2)	0.51786 (12)	0.25306 (9)	0.0642 (4)	
N6	−0.07603 (19)	−0.11995 (14)	0.78182 (12)	0.0498 (3)	
C7	0.2257 (2)	0.49686 (16)	0.78009 (13)	0.0461 (3)	
C8	0.1519 (2)	0.36058 (15)	0.73062 (12)	0.0417 (3)	
H8	0.1090	0.2881	0.7778	0.050*	
C9	0.14239 (19)	0.33355 (13)	0.62055 (11)	0.0348 (3)	
C10	0.21349 (18)	0.44638 (13)	0.54632 (11)	0.0331 (3)	
C11	0.2836 (2)	0.57753 (14)	0.59446 (11)	0.0366 (3)	
C12	0.3533 (2)	0.69027 (14)	0.53032 (13)	0.0432 (3)	
H12	0.3988	0.7768	0.5643	0.052*	
C13	0.3553 (2)	0.67440 (14)	0.41558 (13)	0.0425 (3)	
H13	0.4036	0.7500	0.3721	0.051*	
C14	0.2849 (2)	0.54501 (15)	0.36542 (12)	0.0410 (3)	
C15	0.2157 (2)	0.43231 (14)	0.43028 (11)	0.0380 (3)	
H15	0.1700	0.3460	0.3960	0.046*	
C16	0.3336 (3)	0.62831 (19)	0.18046 (14)	0.0585 (4)	
H16A	0.3220	0.5938	0.1045	0.088*	
H16B	0.4694	0.6806	0.1961	0.088*	
H16C	0.2468	0.6854	0.1916	0.088*	
C17	0.0638 (2)	0.19172 (14)	0.56997 (12)	0.0415 (3)	
H17A	0.1767	0.1563	0.5551	0.050*	
H17B	0.0011	0.1976	0.4988	0.050*	
C18	0.0326 (2)	−0.02097 (14)	0.72018 (11)	0.0380 (3)	
C19	0.0146 (3)	−0.20858 (17)	0.84504 (14)	0.0516 (4)	
H19A	0.1589	−0.1777	0.8384	0.062*	
H19B	−0.0318	−0.3013	0.8143	0.062*	
C20	−0.0410 (3)	−0.2057 (2)	0.96626 (16)	0.0684 (5)	
H20A	0.0082	−0.2715	1.0051	0.082*	
H20B	0.0233	−0.1160	0.9995	0.082*	
C21	−0.2638 (4)	−0.2379 (3)	0.98128 (18)	0.0868 (8)	
H21A	−0.3259	−0.3330	0.9601	0.104*	
H21B	−0.2915	−0.2246	1.0594	0.104*	
C22	−0.3539 (3)	−0.1499 (2)	0.91250 (16)	0.0643 (5)	
H22A	−0.3084	−0.0563	0.9413	0.077*	
H22B	−0.4984	−0.1805	0.9179	0.077*	
C23	−0.2955 (2)	−0.15637 (19)	0.79241 (15)	0.0540 (4)	
H23A	−0.3544	−0.2479	0.7612	0.065*	
H23B	−0.3471	−0.0940	0.7505	0.065*	

### Atomic displacement parameters (Å^2^)

**Table:**

	*U*^11^	*U*^22^	*U*^33^	*U*^12^	*U*^13^	*U*^23^
S1	0.0397 (2)	0.03896 (19)	0.0547 (2)	0.01085 (15)	0.00079 (15)	0.01674 (15)
S2	0.0365 (2)	0.0502 (2)	0.0774 (3)	0.01264 (17)	0.01009 (18)	0.0240 (2)
O3	0.0547 (6)	0.0401 (5)	0.0431 (5)	0.0095 (5)	−0.0055 (5)	0.0000 (4)
O4	0.0994 (11)	0.0649 (8)	0.0402 (6)	0.0193 (7)	−0.0079 (6)	−0.0026 (5)
O5	0.0992 (10)	0.0489 (7)	0.0404 (6)	0.0126 (6)	0.0149 (6)	0.0142 (5)
N6	0.0354 (6)	0.0536 (8)	0.0617 (8)	0.0112 (5)	0.0035 (5)	0.0300 (6)
C7	0.0487 (8)	0.0491 (8)	0.0412 (8)	0.0150 (7)	−0.0044 (6)	0.0037 (6)
C8	0.0443 (8)	0.0421 (7)	0.0403 (7)	0.0140 (6)	0.0002 (6)	0.0095 (6)
C9	0.0323 (6)	0.0349 (6)	0.0395 (7)	0.0126 (5)	0.0008 (5)	0.0081 (5)
C10	0.0286 (6)	0.0332 (6)	0.0399 (6)	0.0120 (5)	0.0011 (5)	0.0075 (5)
C11	0.0321 (6)	0.0367 (7)	0.0424 (7)	0.0114 (5)	−0.0018 (5)	0.0047 (5)
C12	0.0397 (7)	0.0326 (7)	0.0560 (8)	0.0075 (6)	−0.0037 (6)	0.0057 (6)
C13	0.0372 (7)	0.0364 (7)	0.0546 (8)	0.0100 (6)	0.0049 (6)	0.0163 (6)
C14	0.0397 (7)	0.0430 (7)	0.0424 (7)	0.0138 (6)	0.0066 (6)	0.0118 (6)
C15	0.0406 (7)	0.0336 (6)	0.0411 (7)	0.0121 (5)	0.0051 (5)	0.0073 (5)
C16	0.0623 (11)	0.0646 (11)	0.0490 (9)	0.0158 (9)	0.0132 (7)	0.0250 (8)
C17	0.0499 (8)	0.0344 (7)	0.0402 (7)	0.0109 (6)	0.0042 (6)	0.0102 (5)
C18	0.0385 (7)	0.0338 (6)	0.0416 (7)	0.0095 (5)	0.0008 (5)	0.0080 (5)
C19	0.0476 (9)	0.0489 (8)	0.0616 (9)	0.0161 (7)	0.0026 (7)	0.0253 (7)
C20	0.0691 (12)	0.0856 (14)	0.0536 (10)	0.0250 (11)	−0.0086 (9)	0.0175 (9)
C21	0.0761 (14)	0.133 (2)	0.0569 (11)	0.0354 (14)	0.0170 (10)	0.0390 (13)
C22	0.0486 (10)	0.0764 (13)	0.0676 (11)	0.0174 (9)	0.0071 (8)	0.0037 (9)
C23	0.0351 (7)	0.0615 (10)	0.0624 (10)	0.0061 (7)	0.0018 (7)	0.0264 (8)

### Geometric parameters (Å, º)

**Table:**

S1—C18	1.7837 (14)	C13—H13	0.9300
S1—C17	1.7980 (14)	C14—C15	1.3847 (18)
S2—C18	1.6662 (14)	C15—H15	0.9300
O3—C7	1.3697 (18)	C16—H16A	0.9600
O3—C11	1.3774 (17)	C16—H16B	0.9600
O4—C7	1.2008 (19)	C16—H16C	0.9600
O5—C14	1.3647 (18)	C17—H17A	0.9700
O5—C16	1.4169 (18)	C17—H17B	0.9700
N6—C18	1.3293 (17)	C19—C20	1.507 (3)
N6—C19	1.4722 (18)	C19—H19A	0.9700
N6—C23	1.4758 (19)	C19—H19B	0.9700
C7—C8	1.447 (2)	C20—C21	1.507 (3)
C8—C9	1.3370 (19)	C20—H20A	0.9700
C8—H8	0.9300	C20—H20B	0.9700
C9—C10	1.4565 (17)	C21—C22	1.502 (3)
C9—C17	1.503 (2)	C21—H21A	0.9700
C10—C11	1.3942 (19)	C21—H21B	0.9700
C10—C15	1.3949 (18)	C22—C23	1.502 (3)
C11—C12	1.3803 (19)	C22—H22A	0.9700
C12—C13	1.381 (2)	C22—H22B	0.9700
C12—H12	0.9300	C23—H23A	0.9700
C13—C14	1.389 (2)	C23—H23B	0.9700
			
C18—S1—C17	104.68 (7)	C9—C17—S1	116.29 (10)
C7—O3—C11	121.57 (11)	C9—C17—H17A	108.2
C14—O5—C16	118.89 (14)	S1—C17—H17A	108.2
C18—N6—C19	122.03 (13)	C9—C17—H17B	108.2
C18—N6—C23	125.13 (12)	S1—C17—H17B	108.2
C19—N6—C23	112.81 (12)	H17A—C17—H17B	107.4
O4—C7—O3	117.03 (15)	N6—C18—S2	124.86 (11)
O4—C7—C8	126.12 (15)	N6—C18—S1	113.21 (10)
O3—C7—C8	116.85 (13)	S2—C18—S1	121.91 (8)
C9—C8—C7	123.36 (13)	N6—C19—C20	110.31 (15)
C9—C8—H8	118.3	N6—C19—H19A	109.6
C7—C8—H8	118.3	C20—C19—H19A	109.6
C8—C9—C10	118.56 (12)	N6—C19—H19B	109.6
C8—C9—C17	122.94 (12)	C20—C19—H19B	109.6
C10—C9—C17	118.49 (11)	H19A—C19—H19B	108.1
C11—C10—C15	117.71 (12)	C21—C20—C19	112.10 (16)
C11—C10—C9	117.80 (12)	C21—C20—H20A	109.2
C15—C10—C9	124.49 (12)	C19—C20—H20A	109.2
O3—C11—C12	116.55 (12)	C21—C20—H20B	109.2
O3—C11—C10	121.83 (12)	C19—C20—H20B	109.2
C12—C11—C10	121.62 (13)	H20A—C20—H20B	107.9
C11—C12—C13	119.90 (13)	C22—C21—C20	112.04 (17)
C11—C12—H12	120.0	C22—C21—H21A	109.2
C13—C12—H12	120.0	C20—C21—H21A	109.2
C12—C13—C14	119.66 (12)	C22—C21—H21B	109.2
C12—C13—H13	120.2	C20—C21—H21B	109.2
C14—C13—H13	120.2	H21A—C21—H21B	107.9
O5—C14—C15	115.35 (13)	C21—C22—C23	110.75 (17)
O5—C14—C13	124.52 (12)	C21—C22—H22A	109.5
C15—C14—C13	120.13 (13)	C23—C22—H22A	109.5
C14—C15—C10	120.97 (13)	C21—C22—H22B	109.5
C14—C15—H15	119.5	C23—C22—H22B	109.5
C10—C15—H15	119.5	H22A—C22—H22B	108.1
O5—C16—H16A	109.5	N6—C23—C22	110.94 (14)
O5—C16—H16B	109.5	N6—C23—H23A	109.5
H16A—C16—H16B	109.5	C22—C23—H23A	109.5
O5—C16—H16C	109.5	N6—C23—H23B	109.5
H16A—C16—H16C	109.5	C22—C23—H23B	109.5
H16B—C16—H16C	109.5	H23A—C23—H23B	108.0

### Hydrogen-bond geometry (Å, º)

**Table:**

*D*—H···*A*	*D*—H	H···*A*	*D*···*A*	*D*—H···*A*
C19—H19*B*···O5^i^	0.97	2.50	3.410 (2)	157
C23—H23*A*···O3^ii^	0.97	2.60	3.365 (2)	136

Symmetry codes: (i) −*x*, −*y*, −*z*+1; (ii) *x*−1, *y*−1, *z*.
